# DNA damage induces a meiotic arrest in mouse oocytes mediated by the spindle assembly checkpoint

**DOI:** 10.1038/ncomms9553

**Published:** 2015-11-02

**Authors:** Josie K. Collins, Simon I. R. Lane, Julie A. Merriman, Keith T. Jones

**Affiliations:** 1 Centre for Biological Sciences, Faculty of Natural and Environmental Sciences, University of Southampton, Southampton SO17 1BJ, UK

## Abstract

Extensive damage to maternal DNA during meiosis causes infertility, birth defects and abortions. However, it is unknown if fully grown oocytes have a mechanism to prevent the creation of DNA-damaged embryos. Here we show that DNA damage activates a pathway involving the spindle assembly checkpoint (SAC) in response to chemically induced double strand breaks, UVB and ionizing radiation. DNA damage can occur either before or after nuclear envelope breakdown, and provides an effective block to anaphase-promoting complex activity, and consequently the formation of mature eggs. This contrasts with somatic cells, where DNA damage fails to affect mitotic progression. However, it uncovers a second function for the meiotic SAC, which in the context of detecting microtubule–kinetochore errors has hitherto been labelled as weak or ineffectual in mammalian oocytes. We propose that its essential role in the detection of DNA damage sheds new light on its biological purpose in mammalian female meiosis.

## Abstract (web summary)

Damage to maternal DNA during meosis can lead to birth defects, abortion or infertility. Here, the authors show that the spindle assembly checkpoint can respond to DNA damage in oocytes by blocking anaphase promoting complex activity and arresting oocytes in meiosis I.

## Graphical abstract

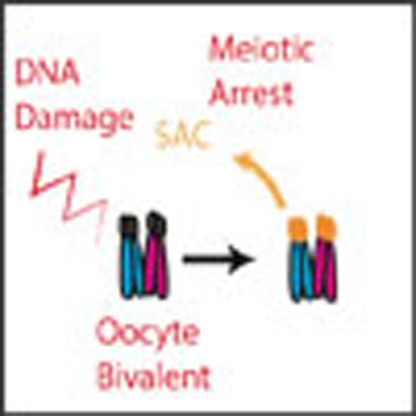

Maternal DNA damage is universally detrimental to reproductive success: causing infertility, birth defects and abortions^1^,^2^,^3^. However, strategies within gametes are known to exist that mitigate the impact of these events^4^. For example in the early phases of follicular growth, oocyte atresia is stimulated following DNA damage^5^. As such, oocytes contained in primordial follicles undergo apoptosis in response to DNA damage, so protecting the propagation of harmful mutations from the female germline. However, this can lead to sterility in women undergoing chemo- or radiotherapy due to a marked reduction in ovarian follicle reserve, and at a cellular level is mediated in oocytes by activation of the p53 family member Trp63 (refs 5, 6). However, Trp63 expression is limited to oocytes from primordial and primary follicles, and as such is absent from fully grown oocytes that are found in more advanced stage follicles^5^. Therefore, given the high protection placed on oocytes in primordial follicles, one would anticipate a p53-independent mechanism in fully grown oocytes that prevents formation of DNA-damaged embryos. However, this supposition appears incorrect in the face of observations that show a weak G2/M transition DNA damage response (DDR) checkpoint and catenation checkpoint in fully grown oocytes^7^,^8^, both of which are prominent in somatic cells^9^,^10^.

The SAC is a universally employed cell checkpoint, present in mouse oocytes^11^,^12^,^13^,^14^,^15^,^16^,^17^, that acts to prevent aneuploidy by inhibiting the activity of the anaphase-promoting complex (APC) before chromosomes are ready to divide faithfully^18^. In somatic cells, the SAC prevents chromatid mis-segregation at anaphase, and consequently aneuploidy in daughter cells, by inhibiting APC activity until a time at which kinetochores from sister chromatid pairs are all attached to microtubules^18^,^19^. The SAC, which is composed principally of members of the Bub and Mad families, does this by using unoccupied kinetochores as a platform to build and amplify a cytosolic and potent APC inhibitory complex^20^. Furthermore, the attachment generates tension across sister chromatids, when the kinetochores of the pair attach to microtubules emanating from opposite poles. This state, known as biorientation, promotes faithful segregation of chromatids at anaphase. Development of tension across the sister chromatid pair aids biorientation by stabilizing microtubule interaction with the kinetochores, so turning weak often side-on interaction into strong end-on k-fibres that can pull on chromosomes^19^,^20^.

We speculated here whether the meiotic SAC could be involved in sensing DNA damage in oocytes, so providing a protective checkpoint against the formation of embryos with damaged DNA. Indeed, neocarzinostatin and laser-beam dissection that cuts DNA, have both been reported to delay or arrest oocytes in meiosis I (MI)^21^,^22^. Furthermore, there is growing evidence to show cross-talk between two major cell cycle checkpoints: the DDR and the SAC^23^,^24^,^25^,^26^,^27^. As such, proteins associated with one checkpoint have been identified as having moonlighting roles in the other. For example, RNAi silencing of nearly all members of the Fanconi Anaemia pathway, involved in DNA crosslink repair, abrogates the SAC in both HeLa cells and primary cells cultured from Fanconi Anaemia patients^26^. Further, in DT40 cells, loss of the DDR kinase Chk1 leads to a failure of the SAC component BubR1 to be targeted to kinetochores, possibly due to lowered activity and phosphorylation of Aurora kinase B, and this is associated with an inability of the cells to arrest with taxol^27^. In addition, ataxia telangiectasia mutated kinase and MDC1 have been shown to be important in recruiting the SAC component Mad2 and the APC activator Cdc20 to kinetochores of U2OS cells^24^. In budding yeast, the reciprocal association has also been reported, with Mad2 involved in a DDR response during the G2/M transition^23^. Similarly, in HeLa cells Bub1 contributes to an optimal DDR following ionizing radiation, and has been shown to be a substrate of ataxia telangiectasia mutated^28^.

Despite the possibilities of overlapping functions for some DDR proteins in the SAC, it seems clear from somatic cells that the DDR is actually suppressed during mitosis^29^,^30^,^31^,^32^. So for example, when human somatic cell lines have their DNA damaged in mitosis they do not activate the SAC^32^. Indeed a diminution in the efficacy of the DDR during mitosis is thought to be essential to protect against telomere fusions that could otherwise be generated during repair^33^. Therefore, the present study set out to establish whether there was meiotic association of DNA damage with the SAC. We find that both physical- and chemical-induced DNA damage given to mouse oocytes has the capacity to induce an MI arrest. This arrest is associated with a block to APC activity and raised levels of Mad2 on kinetochores. Furthermore, it is dependent on the presence of Mad2 and can be alleviated by reducing Mps1 activity. As such it is proposed that DNA damage has the capacity to help prevent the creation of deleterious eggs by activating a pathway involving SAC proteins.

## Results

### DNA damage does not block NEB

**Figure 1:**
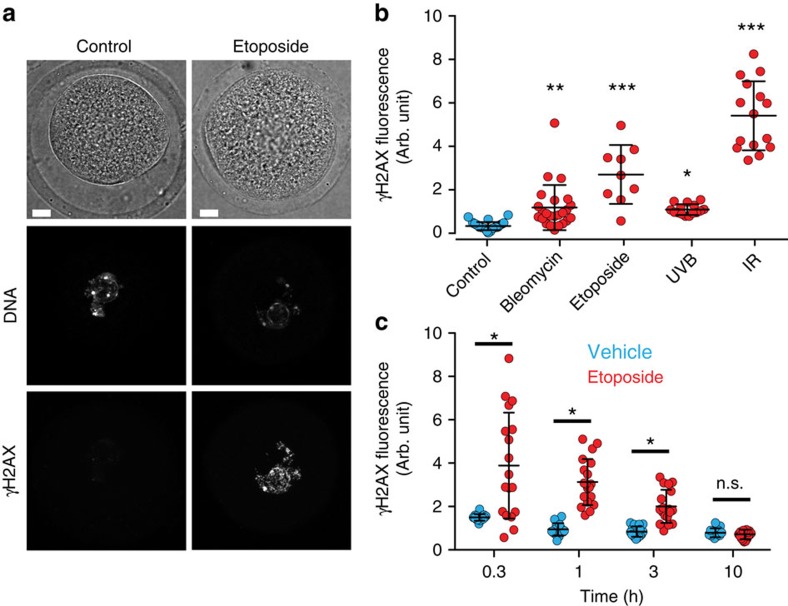
DNA damage induction and repair in GV oocytes. (**a**) Representative γH2AX immunofluorescence in a GV oocyte following either no treatment (control), or etoposide (25 μg ml^−1^). γH2AX staining is negligible in the control oocyte but visible in the nucleus following DNA damage. Scale bar, 5 μm. (**b**) Nuclear γH2AX immunofluorescence levels in individual oocytes following etopiside (25 μg ml^−1^), bleomycin (1 μM), ultraviolet B (UVB, 300 nm, 30 s) or ionizing radiation (IR, 4.5 Gy). Data are pooled from three mice for each condition. (**c**) Nuclear γH2AX accumulation in oocytes at various times after etoposide (25 μg ml^−1^) exposure. Oocytes were maintained in GV arrest for the times indicated after 15-min exposure to etoposide or vehicle (0.1% DMSO). Nuclear γH2AX accumulation following etoposide exposure is initially high, but drops to control levels over 10 h. Oocytes were pooled from four mice. (**a**–**c**) Oocytes were fixed at either 15 min (etoposide and bleomycin) or 40 min after treatment. (**b**,**c**) Each data point represents one oocyte; means and s.d. are represented by the horizontal lines; *P<0.05, compared with control; **P<0.001, ***P<0.0001 (ANOVA, Tukey's *post hoc* test).

To investigate the potential of DNA damage to affect completion of MI, fully grown germinal vesicle (GV) stage oocytes collected from antral follicles were briefly treated with etoposide, bleomycin, ultraviolet B or gamma-ionizing radiation. It is known that all of these agents induce a DDR in somatic cells, as assessed by phosphorylation of histone H2AX (γH2AX), at sites of DNA damage^34^,^35^, and in oocytes γH2AX levels were similarly raised (Fig. 1a,b). With time, γH2AX staining should decrease as the DNA damage is repaired^36^, and it was confirmed that oocytes do have the capacity to do this with respect to one of the DNA-damaging agents, etoposide, in GV oocytes over a period of the next 3–10 h (Fig. 1c).

We wanted to assess the impact on GV oocytes of carrying DNA damage during MI. Normally, meiotic resumption in GV oocytes is initiated ∼1 h following the release from maturation-arresting media. This is initially observed as nuclear envelope breakdown (NEB), which is equivalent to a G2/M transition in somatic cells^37^. Therefore, shortly after DNA damage was induced oocytes were washed free from milrinone to allow them to initiate meiotic resumption^38^,^39^. For all four DNA-damaging agents we observed normal rates and timing of NEB, in agreement with previous studies that have demonstrated mouse oocytes are unable to mount a robust G2 arrest in response to DNA damage^7^. For three of these agents (ultraviolet B, bleomycin and etoposide), where we could perform timelapse imaging immediately following DNA damage, we went on to confirm the lack of effect on NEB. We measured the timing of NEB, and found that DNA damage altered the mean timing at which 50% of the oocytes underwent NEB by a matter of 10 min at most (Supplementary Fig. 1).

### DNA damage to GV oocytes induces an MI arrest

**Figure 2:**
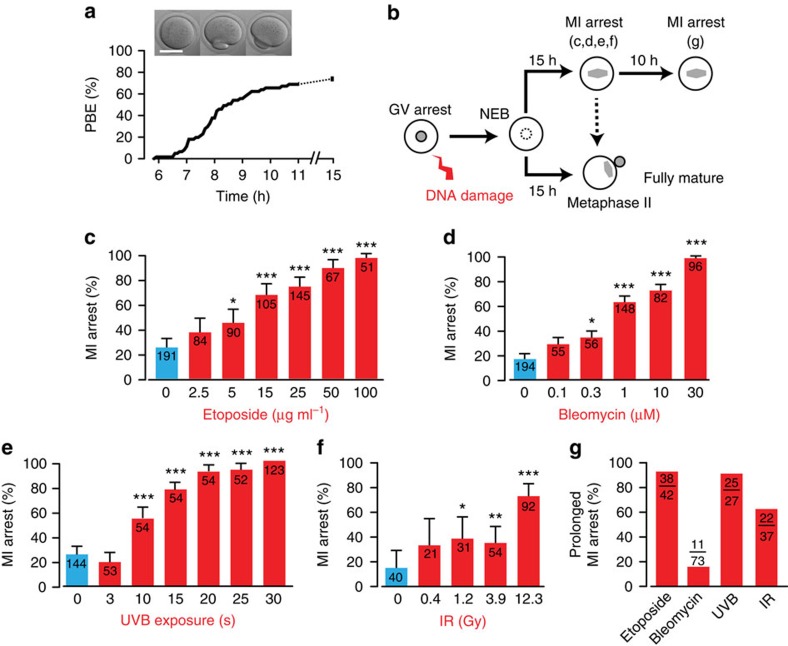
DNA damage during GV arrest induces an MI arrest. (**a**) PBE in untreated oocytes at the times indicated after NEB. The majority of oocytes complete MI between 8 and 11 h after NEB. Data are from 59 oocytes, pooled from three mice. Images of PBE, scale bar, 50 μm. (**b**) Schematic of the experimental design to assess the effects of DNA damage on meiotic progression. (**c**–**f**) MI-arrest rates in response to DNA damage that was induced during GV arrest with various doses of etoposide (**c**) bleomycin (**d**) ultraviolet B (UVB) exposure at 300 nm (**e**) or ionizing radiation (IR) (**f**). Arrest was assessed at 15 h. (**g**) Prolonged MI arrest rate at 25 h after NEB. Oocytes that were initially MI arrested at 15 h, following GV-stage DNA damage (etoposide 25 μg ml^−1^, bleomycin 10 μM, ultraviolet B exposure 15 s, ionizing radiation 12.3 Gy), were further cultured for 10 h and assessed for PBE. (**c**–**g**) Numbers of oocytes used are as indicated (pooled from between two and six mice per treatment). *P<0.05, compared with ‘0'; **P<0.001, ***P<0.0001 (Fisher's exact test). Note: s.e. is absent when arrest was 100% for all oocytes examined.

Over the course of the next 11 h following NEB (all timings are given relative to NEB) untreated oocytes complete MI; this reductional division ends with the separation of the homologous chromosomes pairs (bivalents), and can be measured by the formation of the first polar body (polar body extrusion; PBE; Fig. 2a)^40^. GV oocytes exposed to ionizing radiation or ultraviolet B, or treated with etoposide or bleomycin were allowed to undergo MI following DNA damage, and were assessed for PBE at 15 h (Fig. 2b). This time is 4 h after oocytes have normally completed MI and undergone PBE. We observed that all DNA-damaging agents caused a dose-dependent block to MI completion (Fig. 2c–f). To determine if this inability to undergo PBE was a block or delay in MI, oocytes were assessed a further 10 h later. Following ionizing radiation, etoposide or ultraviolet B, but not bleomycin, the vast majority of oocytes that had been in MI at 15 h also failed to undergo PBE at 25 h (Fig. 2g). It is concluded therefore that for some oocytes treated with etoposide, and for most oocytes damaged by ionizing radiation, etoposide or ultraviolet B, there is a very effective block to progression through MI.

### DNA damage during MI induces an MI arrest

**Figure 3:**
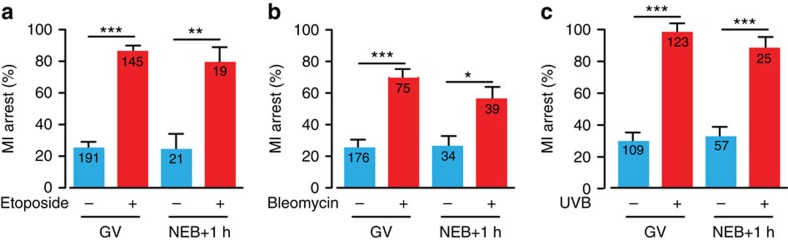
DNA damage following NEB causes MI arrest. (**a**–**c**) MI-arrest rates in oocytes that followed DNA damage induction either during GV arrest or 1 h after NEB; (**a**) etoposide addition (25 μg ml^−1^), (**b**) bleomycin (1 μM); and (**c**) ultraviolet B exposure (300 nm, 30 s). Numbers of oocytes used are as indicated (pooled from between 2 and 6 mice per treatment); (**a**,**b**) compared against vehicle addition; (**c**) no exposure. *P<0.05; **P<0.001, ***P<0.0001 (Fisher's exact test); (**a**–**c**) error bars are s.e.'s.

For all four DNA-damaging agents, the protocol above involved their addition during GV arrest, which is equivalent to G2 in mitotic cells. For bleomycin, etoposide and ultraviolet B (but not ionizing radiation due to practical constraints), we also checked if DNA damage had the ability to arrest oocytes in MI if administered following NEB at time equivalent to M-phase. We observed that all three agents did this, and as such DNA damage does not have to occur before meiotic resumption in order to cause an arrest (Fig. 3). Therefore, contrary to somatic cells where DNA damage in M-phase fails to cause any arrest^32^, oocytes seem able to stall their meiotic division quite effectively when DNA damage is induced during the MI division.

### DNA damage generates fragments but normal MI spindle forms

**Figure 4:**
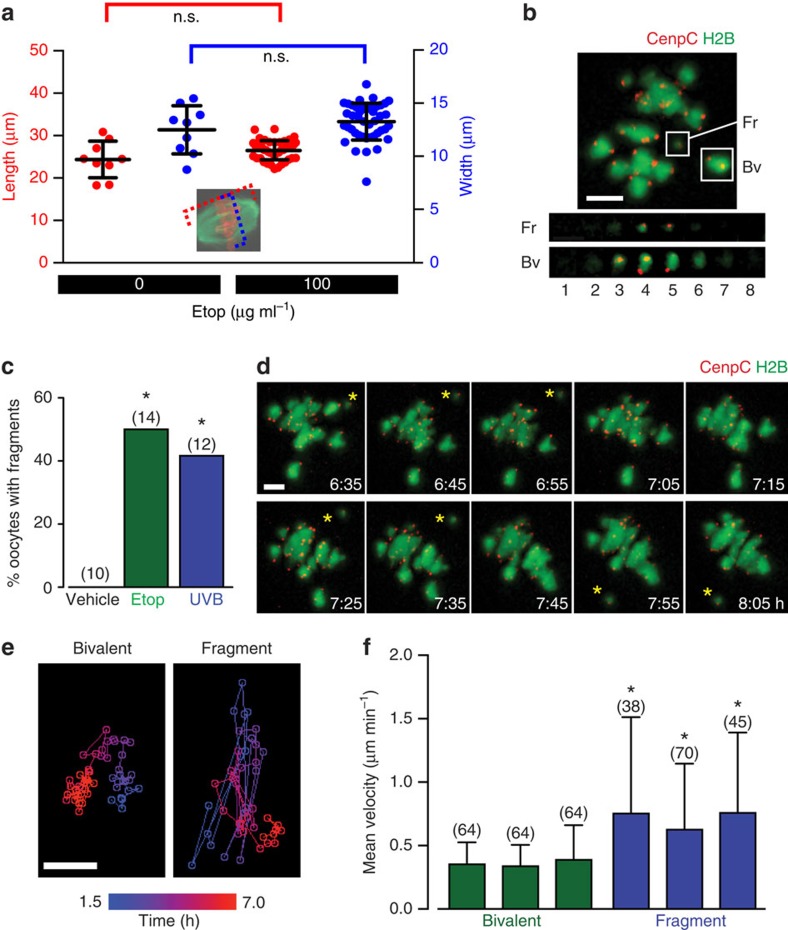
DNA damage permits spindle formation but causes DNA fragmentation. (**a**) Meiotic spindle measurements of length and width taken on fixed oocytes, following addition of either vehicle or etoposide for 15 min during GV arrest. Neither spindle length nor width was affected by etoposide addition (n.s., *P*>0.05, ANOVA with Tukey's *post hoc* analysis). Between 9 and 45 oocytes were analysed per group, with oocytes pooled from six mice, displayed are means with s.d. (**b**) A chromosome fragment (Fr) generated in an oocyte following etoposide addition (25 μg ml^−1^). This fragment possesses a single kinetochore pair, seen by counting kinetochores in the individual *z*-slices (numbered 1–8). For comparison, the two sister kinetochore pairs of a bivalent are also illustrated (Bv). (**c**) Percentage of etoposide- (25 μg ml^−1^) or ultraviolet B (300 nm, 15 s)-treated oocytes, expressing CenpC and H2B, found to have bivalent fragments during 4D CLSM. Images were captured every 10 min over a period of 15 h. Numbers of oocytes used are indicated (data pooled from two mice for each condition). The rate of fragmentation was precisely measured in each oocyte, and not under-reported, by counting for the expected number of kinetochores (40 CenpC foci) and ensuring that no DNA fragments were outside of the imaging volume. *P<0.05 Fisher's exact test. (**d**) Timelapse of an oocyte expressing CenpC and H2B, with a single kinetochore fragment (*). Note its erratic movement around the spindle equator. (**e**) Positional plots of a bivalent and a DNA fragment moving in an oocyte at the times indicated during MI. The fragment moves more erratically around the spindle than the bivalent. (**f**) Velocity (mean with s.d.) of bivalents and fragments in DNA-damaged oocytes. *, significantly different from every bivalent group, *P*<0.05 (ANOVA, Tukey's *post hoc* test). (**b**,**d**,**e**) Scale bar, 5 μm.

To fully understand how and why DNA-damaged oocytes arrest in MI, we looked at spindle morphology and the bivalents themselves during MI. For all four DNA-damaging agents, we did observe what appeared to be a normal metaphase plate of chromatin in those oocytes that remained MI arrested. To explore this in more detail, we fixed those oocytes that had been treated with etoposide, and further stained for tubulin to measure spindle morphology. In such oocytes, a morphologically normal, barrel-shaped meiotic spindle, was observed with dimensions indistinguishable from time-matched controls (Fig. 4a).

Given that exogenously introduced DNA-coated beads have the capacity to congress with endogenous chromosomes on a meiotic spindle in mouse oocytes^41^, the morphologically normal spindle gives little insight into the level of DNA fragmentation that may have taken place. Therefore, given that all these agents have a capacity to fragment DNA and so induce double-strand breaks (DSBs), we anticipated that breakage could result in the generation of bivalent fragments if multiple DSBs had occurred in the same bivalent. We used etoposide as a chemical method of inducing DSBs, and UV, as a physical method, to cause MI arrest; and examined for the presence of DNA fragments following NEB (Fig. 4b). Fragments were found in 40–50% of oocytes examined (Fig. 4c), and we confirmed that the rate of fragmentation was precisely measured in each oocyte, and not under-reported, by counting for the expected number of kinetochores (40 CenpC foci in each oocyte) and for any cortical DNA fragments outside of the imaging volume at the completion of the experiment. The bivalent fragments were further characterized, and distinguished from bivalents, by their erratic oscillations across the spindle equator (Fig. 4d), and their greater speed (Fig. 4e,f). This would be consistent with the fragments containing a single kinetochore pair, which develops only temporary interactions with spindle microtubules that are continually being destabilized due to the lack of tension.

We questioned whether DNA fragments were responsible for the MI arrest. However, two lines of evidence suggested this was not the case. First, 50–60% of oocytes, expressing H2B and CenpC, that were DNA damaged with ultraviolet B or etoposide at the GV stage, and arrested at MI, contained no fragments during the course of MI (Fig. 4c). Second, it has been shown previously that a small number of univalents or non-biorientated bivalents have no ability to arrest oocytes in MI^11^,^12^,^13^,^14^,^15^,^42^.

### Biorientation is not affected by DNA damage

**Figure 5:**
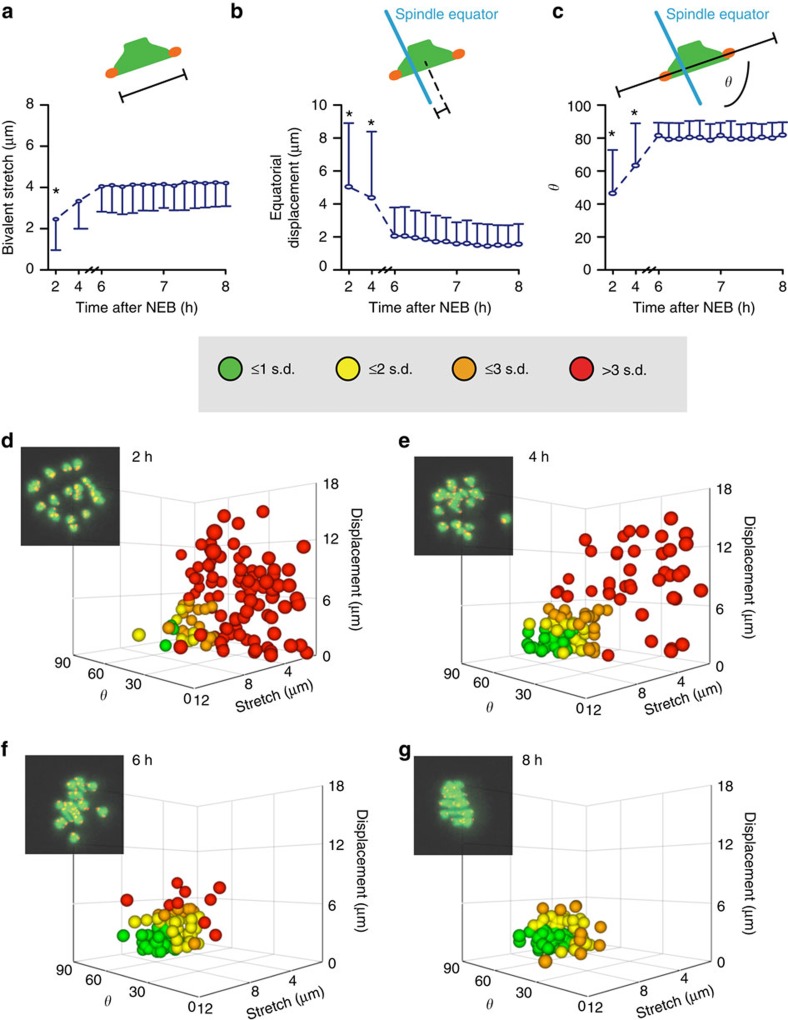
Bivalent congression measured by live cell imaging. Live imaging of untreated oocytes expressing H2B-mCherry and CenpC-GFP. (**a**–**c**) 120 bivalents (*n*=6 oocytes) completing MI, transitioned from an initial state of non-congression (2 h) to a highly congressed state (6–8 h). The three parameters measured were: the distance apart of sister kinetochore pairs (a; bivalent stretch); the distance of the bivalent center from the spindle equator (b; displacement); and, the angle of intersection between the bivalents kinetochores and the spindle equator ((**c**) *θ*). Plotted are means and s.d., * indicates significant difference from other timepoints, *P*<0.05, ANOVA with Tukey's *post hoc* test. (**d**–**g**) The measurements as shown in (**a**–**c**) were plotted on the same axes for individual bivalents at four timepoints throughout MI ((**d**) 2 h; (**e**) 4 h; (**f**) 6 h; (**g**) 8 h). Data points are coloured according to how many s.d.'s they lie from the mean value for each parameter at 8 h. The greatest s.d. from the mean for any of the three measurements defines the colour of the bivalent. Insets show a *z*-projection of one oocyte from the corresponding timepoint. CenpC, red; H2B, green.

**Figure 6:**
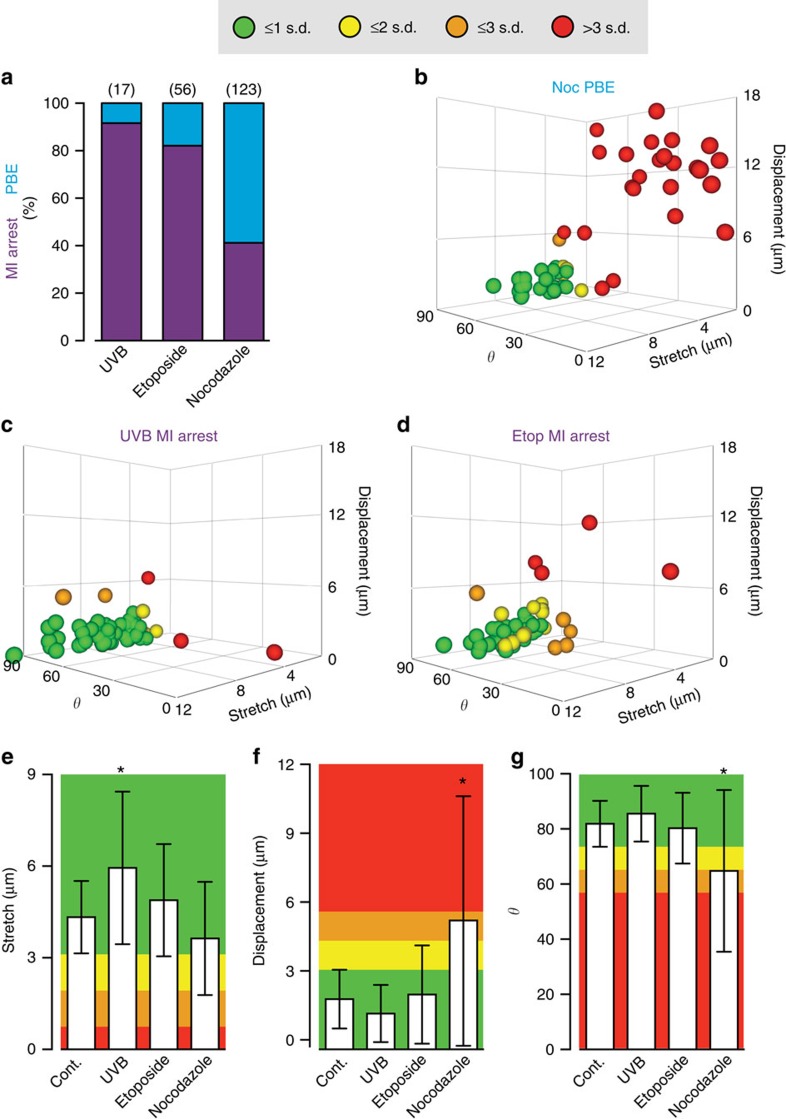
MI arrest following DNA damage is independent of bivalent biorientation. (**a**) Percentage of oocytes that undergo PBE or MI arrest following GV-stage treatment with ultraviolet B (15 s), etoposide (25 μg ml^−1^, 15 min), or a low dose of nocodazole (25 nM) added for the duration of MI. (**b**–**d**) Bivalent tension (stretch), displacement from the metaphase plate (displacement) and bivalent orientation (*θ*); in oocytes treated throughout MI with nocodazole (**b**) or before NEB for 15 s with ultraviolet B (**c**) or 15 min with etoposide (**d**). Data points are individual bivalents combined from three oocytes per treatment group at 8 h. The maximum number of s.d.'s from the 8 h PBE group mean on any axis is used to colour the bivalent. The three nocodazole-treated oocytes all underwent anaphase in the next 10 min. (**e**–**g**) Bivalent stretch (**e**) displacement (**e**) and orientation (**f**) from untreated oocytes and those in (**b**–**d**). Each group assessed 3 oocytes and 60 bivalents. Bars indicate means, and errors are s.d. Background colouring represents multiple s.d.'s from the mean in the control group. *, indicates significant difference from control, *P*<0.05, ANOVA with Tukey's *post hoc* test.

We speculated that if fragments *per se* were not the cause of the arrest, maybe bivalent congression, or tension development across biorientated bivalents were being perturbed by DNA damage. Using three-dimensional position logging of sister kinetochore pairs^43^,^44^,^45^, three parameters that ascribe congression and biorientation were measured: (i) bivalent tension, measured as the distance between sister kinetochores pairs, which increases when bivalents become under amphitelic attachment from microtubules emanating from opposite poles (that is, biorientation; Fig. 5a); (ii) displacement from the metaphase plate, which reduces as balanced microtubule forces position bivalents at the spindle midpoint (Fig. 5b); and, (iii) bivalent orientation, the angle (*θ*) that subtends the axes formed between the bivalent and the spindle equator - and tends to 90° as bivalents align with the pole-to-pole axis (Fig. 5c). A three-axis plot of tension, displacement and *θ*, and colouring each bivalent by the number of s.d.'s from the mean at 8 h, demonstrates the process of congression and biorientation in the hours leading up to anaphase in untreated oocytes (2 h, Fig. 5d; 4 h Fig. 5e; 6 h, Fig. 5f; and 8 h, Fig. 5g).

To determine if the bivalent measurements of stretch, displacement and *θ* observed in DNA-damaged oocytes could be associated with MI arrest, we compared them with the same measurements taken from oocytes treated with a low dose of the spindle poison nocodazole. We chose a low dose that only mildly raised MI arrest rates (Fig. 6a), and compared 60 bivalents (*n*=3 oocytes) in nocodazole-treated oocytes immediately before anaphase, versus time-matched MI-arresting DNA-damaged oocytes (etoposide and ultraviolet B). We used etoposide as a chemical method of inducing DSBs, and UV, as a physical method. It was anticipated that this low dose of nocodazole would perturb the interactions of microtubules with kinetochores sufficiently to disrupt bivalent biorientation and congression. Indeed, it was observed that immediately before anaphase in nocodazole-treated oocytes many bivalents were not congressed, with distances of up to 18 μm away from the mean bivalent position (an oocyte is 70 μm in diameter; Fig. 6b). In contrast, the vast majority of bivalents in ultraviolet B- (Fig. 6c) and etoposide (Fig. 6d)-treated oocytes showed much greater levels of congression and biorientation, and yet still arrested at MI. Bivalent stretch (Fig. 6e), displacement (Fig. 6f) and alignment (Fig. 6g) all showed consistently better values in DNA-damaged oocytes that went onto arrest, than the same measurements made in nocodazole-treated oocytes that completed MI (Fig. 6e–g). We do note that although biorientation did develop, bivalents in ultraviolet B-damaged oocytes stretched more than undamaged bivalents (Fig. 6c), possibly as a result of ultraviolet B-induced alterations in the physiochemical properties of chromatin. We observed that in nocodazole-treated oocytes, oocytes could readily complete anaphase with as many as eight bivalents that were not biorientated and under tension (Supplementary Fig. 2).

Given that the DNA-damaged oocytes here achieved much better bivalent biorientation when compared with the nocodazole-treated group, and yet remained MI arrested, it seems unlikely that a lack of biorientation is a driver of meiotic arrest. Indeed, we and others have previously found that in mouse oocytes bivalent biorientation appears not to be an essential event for anaphase, as oocytes with severely poorly aligned bivalents, nonetheless, readily undergo separation of their bivalents^11^,^12^,^13^,^14^,^15^. In summary, DNA fragments can be induced by DNA damage, but bivalent integrity is mostly preserved. Therefore, DNA fragments are not needed to induce an MI arrest in response to the damage, and furthermore tension develops across DNA-damaged bivalents, suggesting that the ability of the kinetochores to interact with microtubules is not compromised.

### Mps1 inhibition overcomes DNA-damage-induced arrest

**Figure 7:**
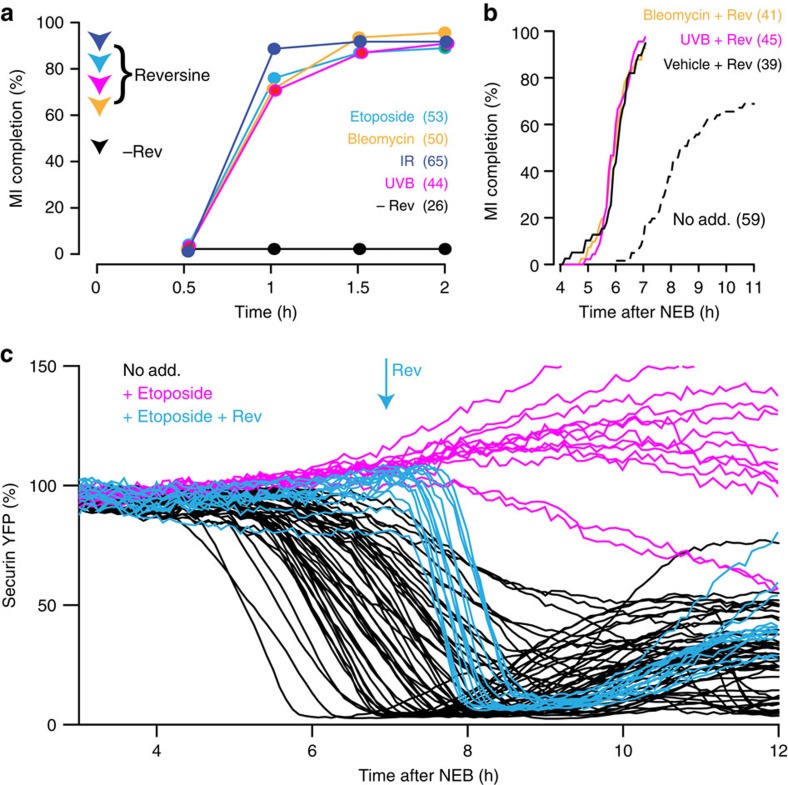
Loss of Mps1 activity overcomes DNA-damage-induced MI arrest. (**a**) MI completion rates following addition of the Mps1 inhibitor reversine (100 nM) to oocytes, at 11 h after NEB, that had been arrested in MI through various methods of DNA damage (etoposide 25 μg ml^−1^; bleomycin 1 μM; ultraviolet B, 15 s; ionizing radiation, 1.2 Gy). Numbers of oocytes used are indicated (data pooled from three to four mice). As a control, vehicle was added to bleomycin-treated oocytes, and all of these remained MI arrested. (**b**) Cumulative MI completion rates for oocytes treated with bleomycin (1 μM), ultraviolet B (300 nm, 15 s) or vehicle (0.1% DMSO) before NEB, and then cultured in the presence of 100 nM reversine (solid lines), or control oocytes without any treatment (dashed line). (**c**) Securin-YFP degradation measured relative to fluorescence at 3 h, in either non-DNA damaged or DNA damaged (etoposide 25 μg ml^−1^ for 15 min during GV arrest). Individual oocytes are recorded, pooled from at least two mice.

To determine if the meiotic arrest induced by DNA damage was SAC mediated, oocytes were treated with the Mps1 inhibitor reversine^46^. For all DNA-damage-inducing agents used, etoposide, bleomycin, ultraviolet B and ionizing radiation, exit from MI arrest was stimulated by reversine addition (Fig. 7a). As a control for the actions of this inhibitor, we showed that reversine had no effect on the meiotic arrest of metaphase II eggs, which are CSF (cytostatic factor) rather than SAC arrested^47^ (Supplementary Fig. 3). Therefore, spontaneous MI arrest was due to SAC-mediated APC inhibition; a checkpoint that was released by the Mps1 inhibitor reversine.

Further to the above, addition of reversine to culture media throughout maturation caused a near-identical acceleration in the timing of PBE in both DNA-damaged oocytes (bleomycin, as a chemical method of inducing DSBs; and ultraviolet B, as a physical method) and vehicle controls (Fig. 7b). This suggested an inability of DNA damage to induce a delay or arrest in MI following abrogation of the SAC. In addition, we confirmed the role of the SAC in the DNA-damage-induced MI arrest, by expressing securin coupled to yellow fluorescent protein (YFP), to measure APC activity, etoposide treating, and then measuring the loss of this APC substrate during the course of oocyte maturation. APC activity was observed to increase nearly 10-fold following reversine addition, while in those oocytes that were DNA damaged securin levels continued to rise, or showed only a very modest loss of securin (Fig. 7c). These measurements all suggest that arrest is mediated by insufficient APC activity, consistent with an active SAC.

### DNA damage arrest is Mad2 dependent

**Figure 8:**
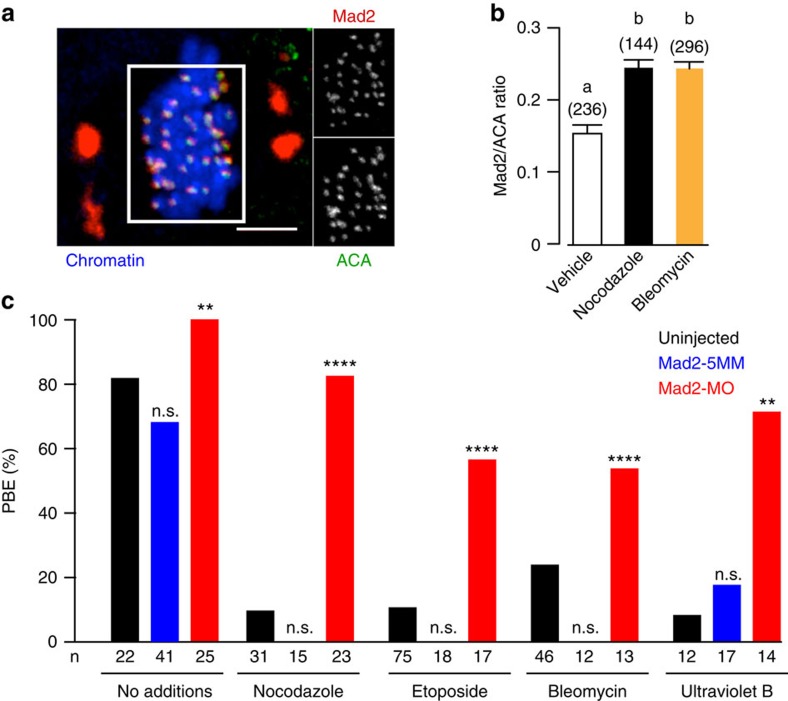
Mad2 needed for DNA-damage-induced MI arrest. (**a**,**b**) Mean kinetochore Mad2 levels in maturing oocytes at 7 h. Oocytes were either treated with nocodazole (400 nM) 15 min before fixation, or with bleomycin (1 μM) or vehicle control during GV arrest. Mad2 immunofluorescence, which could be observed on kinetochores and spindle poles (**a**) was quantified at the kinetochore, and expressed as ratio of fluorescence against anti-centromeric antibody (ACA). Data is pooled from a total of three mice per condition. Different letters signify *P*<0.05 (ANOVA); bars are means with s.d. (**a**) Scale bar, 10 μm. (**c**) Polar body extrusion rate in oocytes, either non-injected, or microinjected with Mad2-MO or Mad2-5MM (5-base mismatch) at the GV stage, and then treated with either no additions, nocodazole (50 nM), etoposide (25 μg ml^−1^), bleomycin (1 μM) or ultraviolet B (UVB, 15 s) as indicated. Numbers of pooled oocytes used are as indicated, with two to four mice used per condition. n.s., non-significant, **P<0.01, ****P<0.0001 (compared with respective uninjected control; Fisher's exact test).

The kinetochore acts as a platform to recruit Mad2 from the cytoplasm, and this forms an essential step in establishment and maintenance of the SAC in inhibiting the APC^18^,^19^,^20^. As oocytes progress through MI, kinetochore-associated Mad2 does decrease, but does not disappear^44^. Instead, this residual Mad2 is associated with some remaining SAC activity that has been measured to reduce the ability of APC to degrade securin by a half, and so extend the duration of MI by about 2 h (ref. 44). It was possible that the MI arrest observed in the DNA-damaged oocytes was also associated with an elevated level of Mad2 on kinetochores. This would be consistent with the signalling pathway employed by the SAC and the measured reduction in APC activity. Kinetochore Mad2 levels were elevated by addition of nocodazole, as a positive control, and compared against time-matched oocytes that had been DNA damaged using bleomycin. Both agents caused a nearly twofold, elevated level of kinetochore Mad2 as assessed by immunofluorescence (Fig. 8a,b). These findings help to establish that the SAC is needed for the DNA damage arrest, and points to a signalling cascade involving the kinetochore.

To confirm the involvement of Mad2 we knocked down levels of this SAC component using an antisense morpholino (Mad2-MO)^48^; and employed a 5-base mismatch morpholino (Mad2-5MM) as control. To DNA-damaged oocytes, we used two chemical methods, etoposide and bleomycin and one physical method, ultraviolet B. In Mad2-5MM microinjected oocytes, or those uninjected, arrest at MI followed DNA damage or nocodazole addition. However, loss of Mad2, through the actions of Mad2-MO caused a rescue in maturation rates for all of these arresting agents (Fig. 8c). These Mad2 knockdown experiments along with the actions of reversine, the measurements of APC activity, and the Mad2 immunofluorescence, provide several independent lines of evidence implicating the SAC in the DNA-damaged-induced MI arrest.

## Discussion

The present study shows that DNA-damaging agents have the ability to activate the SAC in mouse oocytes during MI. These conclusions are based on the findings that (i) the arrest is associated with securin stabilization, a process consistent with APC inhibition, (ii) Mps1 inhibition recovers the ability to initiate securin degradation and complete MI, and, (iii) the arrest is Mad2 dependent. Due to the fact that both Mps1 and Mad2 are fundamental components of the SAC^18^,^19^,^20^, whose downstream target is the APC, we implicate this checkpoint in the DNA-damage-induced meiotic arrest. The timing of DNA damage here was performed primarily prior to NEB, although we observed it can be induced after NEB with the same effect. The present findings used four agents capable of inducing DSBs (chemical damage with bleomycin and etoposide; and physical damage with ultraviolet B and ionizing radiation), but the phenomenon may be independent of the initiating agent, given that neocarzinostatin and laser-beam dissection, which cuts DNA, have previously been reported to delay oocytes in MI or arrest them^21^,^22^. The failure to complete MI appeared to be associated with a long-lasting arrest for three of the four DNA-damaging agents (etoposide; ionizing radiation; ultraviolet B), rather than a small delay, given most undamaged oocytes have undergone MI completion by 9 h, but DNA damage caused substantial numbers to remain arrested until 25 h. During this window, oocytes would have been ovulated (10–11 h) and fertilized (15–18 h)^49^,^50^, with oocytes more than 10 h after ovulation showing greatly reduced ability to become fertilized and produce viable embryos^50^.

It seems likely that a kinetochore-based SAC activation pathway is activated by DNA damage, because Mad2 kinetochore levels were raised. In addition, however, it remains possible that DNA fragments, generated by the four agents used, also contribute to APC inhibition by providing a platform on which the SAC signal is initiated. Indeed, components of the SAC have been shown to assemble independently of the kinetochore^51^,^52^. We only observed fragments in ∼50% of the oocytes examined, so if they do contribute to the SAC-mediated arrest, their size must be below the limits of detection, or they become entangled with other bivalents.

How Mad2 could accumulate on the kinetochores of DNA-damaged oocytes is presently unclear. Bivalents and the their sister kinetochore pairs were tracked by 4D-CLSM during MI following DNA damage to measure accurately the timing of tension development as they became stretched. Such measurements of biorientation were found to be largely unaffected by damage. Therefore, it seems unlikely that DNA damage is affecting tension development across the bivalent. Furthermore, previous studies have highlighted that high levels of bivalent misalignment and lack of tension can be tolerated in oocytes^11^,^12^,^13^,^14^,^15^,^42^. As such it seems highly unlikely that it is a failure of tension development across DNA-damaged bivalents that would raise Mad2 levels on kinetochores sufficient to stall MI. We currently do not fully understand the factors that contribute to the strength of the SAC signal in oocytes^53^,^54^. So, for example, the SAC signal stays partially on during much of MI, reducing APC activity by 50%, but the mechanism behind this rise is not known^44^. Similarly, the level of centromeric cohesin can affect the strength of the SAC signal in mouse oocytes, such that loss of cohesin negatively impacts on the strength of the SAC, although mechanistically how this is achieved remains unknown^55^. This latter finding illustrates an important precedent that factors independent of the immediate location of the kinetochore can influence the strength of the SAC signal during MI. We speculate that DNA damage is similarly a factor able to act at a distance to the kinetochore, and amplify by a process not yet resolved the residual SAC signal that stays partially on in MI^44^.

In somatic cells, the DDR is suppressed during mitosis^29^,^30^,^31^,^32^. Such a diminution in the efficacy of the DDR protects against telomere fusions that could otherwise be generated during repair^33^. Furthermore, and in contrast to the present findings in meiosis, when examined directly in human somatic cells lines, mitotic DNA damage has no capacity to activate the SAC^32^. The meiotic arrest observed in the present study was initiated by both chemical- and physical-damaging stimuli, and is distinct from the M-phase decatenation arrest, which is both SAC- and DNA-damage independent^56^. Furthermore, oocytes lack both a G2/M transition DDR checkpoint and a catenation checkpoint^7^,^8^. Therefore, here we have demonstrated what we believe to be a distinct and novel meiotic checkpoint. The existence of such a DNA checkpoint uncovers a hitherto unappreciated function of the SAC in female mammalian meiosis, which would very much help prevent oocytes with damaged DNA progressing to become fertilizable mature eggs. Future work needs to establish the precise mechanism at play between DNA damage and the SAC, but as a working hypothesis it may involve another member of the p53 family (that is, not Trp63), possibly p73, which is known to be involved in DDR^57^,^58^, but which also seems essential for the establishment of the SAC in mouse oocytes^59^.

The present findings point to the SAC being an important control mechanism in oocytes-preventing formation of DNA-damaged embryos; and allude to a SAC function that extends beyond its recognized actions in monitoring bivalent alignment. The meiotic SAC appears inherently weaker than its mitotic counterpart to the canonical signals of microtubule attachment and tension. With respect to attachment, this may be due to the changes in the architecture of microtubule–kinetochore interaction, which is uniquely set up so as to mono-orientate the sister kinetochore pair in MI, rather than biorientate it as it does in mitosis. With respect to tension, this may be because tension uniquely in MI has to propagate along the length of the chromosome, and therefore would become influenced by the elastic buffering capacity of chromatin. In contrast, the protracted length of meiosis (several hours in mouse) compared with mitosis, may allow a DNA-damage signal to propagate for long enough to influence the SAC.

## Methods

All chemicals and reagents were from Sigma Aldrich (UK) unless otherwise stated.

### Animals and oocyte culture

All mice were used in accordance with the local and UK government regulations on the use of animals in research. Three-to-four-week old C57Bl6 female mice (Charles River, UK) were used. GV-stage oocytes were released from the ovaries of hormonally primed females 44–52 h following 10 IU PMSG-Intervet intraperitoneal injection (Centaur Services, UK). M2 medium supplemented with milrinone (1 μM) was used for collection and to maintain prophase arrest^43^. Oocytes were mechanically stripped from the surrounding cells. For maturation, GV oocytes were washed into fresh M2 media and cultured usually for 14–16 h in M2 or MEM (Amsbio, UK) media, but in some experiments where stated up to 25 h after NEB^43^,^44^.

When needed, etoposide or bleomycin sulphate (Abcam, UK) were added to GV-stage oocytes for 15 min. All drugs were dissolved in dimethylsulphoxide (DMSO) and used at dilutions of 0.1% or below. GV oocytes were also exposed to ionizing irradiation using a Gammacell 1000 Elite (Nordion International Inc., ON, Canada) with a dose rate of 858 cGy min^−1^. Following these various treatments, oocytes were washed free of milrinone immediately unless stated otherwise, or fixed for immunofluorescence. In experiments requiring SAC inhibition, oocytes were allowed to mature for 11 h then reversine (100 nM) added, and polar body extrusion was scored by timelapse microscopy, or by eye at 30 min intervals.

### Immunofluorescence

Oocytes were fixed for 30 min in PHEM (PIPES, HEPES, EGTA, MgCl_2_) buffer containing 2% formaldehyde and 0.05% Triton-X, and were then permeabilized for 15 min in PBS containing 1% PVP and 0.05% Triton-X^11^. Fixing and permeabilizing was performed at room temperature and oocytes were extensively washed with PHEM buffer between solutions. Oocytes were incubated at 4 °C overnight in a blocking buffer of 7% goat serum PBS supplemented with Tween-20 before primary antibody incubation (rabbit anti-γH2AX, Abcam, cat. no. ab11174, 1:200; mouse anti-γ tubulin, Life Technologies, UK, cat. no. A1126, 1:400; ACA, Bioclone, Australia 1:400, cat. no. 90CCS1058; and Mad2, 1:1000, a kind gift from Dr R.H. Chen, Taipei, Taiwan)^11^. Following several washes, oocytes were incubated with secondary antibodies Alexa 633- (1:1000; anti-mouse, cat. no. A21050; anti-rabbit A21070; Life Technologies), 555- (1:1000, cat. no. A21433, Life Technologies) and 488-conjugated (1:1000, cat. no. A11008, Life Technologies). These incubations were at 37 °C in blocking solution. Oocytes were briefly counterstained with Hoechst (20 μg ml^−1^) to label chromatin before being mounted on glass slides with Citifluor (Citifluor Ltd, UK).

### cRNA manufacture

cRNA was transcribed *in vitro* from purified linear dsDNA templates. mMessage T7 RNA kits (Ambion, Life Technologies, UK) or T3 RNA polymerase kits were used for *in vitro* transcription reaction^11^. cRNA was suspended in nuclease-free water and the concentration of RNA products were determined by photospectroscopy.

### Microinjection

Microinjections into oocytes were performed on the stage of an inverted TE300 microscope (Nikon, Japan), using micromanipulators (Narishige, Japan) and a 37 °C heated chamber (Intracel, UK)^43^. A single injection with a 0.1–0.3% volume was achieved using timed injection on a Pneumatic Picopump (World Precision Instruments, UK) and pipette RNA concentrations of 100–500 ng μl^−1^ (refs 11, 43).

### Morpholinos

Mad2 (5′-ATGGCACAGCAGCTCGCCCGAGAGC-3′) and Mad2 5-base mismatch (ATGGC**G**C**T**GCAGCTC**T**CCCG**G**GAGC) morpholinos (Gene Tools LLC, USA) that were first used in a previous study^48^, were diluted in water, and introduced into oocytes by microinjection at tip concentrations of 1 mM. Micropipettes were inserted into cells by using a brief pulse of the negative capacitance overcompensation facility on an electrometer (World Precision Instruments). A precise, bolus injection corresponding to 0.1–1% of the total cell volume was achieved by using a Pneumatic PicoPump (World Precision Instruments). Oocytes were incubated in MEM media with 5% CO_2_ for 24 h to allow for protein knockdown.

### Immunofluorescence imaging

All images were acquired using a Leica SP8 fitted with hybrid detectors and × 63 oil immersion lens. Fluorochromes were imaged sequentially. When quantifying levels of γH2AX, a *z*-stack of the nuclear region was taken (∼30 μm) and acquisition settings were not altered throughout the experiment. γH2AX staining was calculated as total nuclear fluorescence, on an 8-bit scale, following background subtraction from a cytoplasmic region of equal area in the same oocyte. Spindles were imaged with a *z* axis resolution of 2 μm.

### Time-lapse imaging

Timepoints were acquired at 5 or 6 min intervals using a Leica SP8 fitted with hybrid detectors, an environmental chamber set to 37 °C, and a × 40 oil immersion lens. In-lab software written in python programming language was used to image multiple stage regions and to track up to 30 oocytes in experiments using H2B and CenpC to ensure chromosomes stayed in the centre of a 32 × 32 × 32 μm imaging volume.

### Data analysis

All images were processed using Image J (NIH, USA) with extended functionality provided by in-house macros^43^,^44^. To determine nuclear γH2AX, nuclear fluorescence intensity was subtracted from a cytoplasmic background reading for each oocyte. For securin-YFP time-lapse experiments, fluorescence intensities (arbitrary units on an 8-bit scale) were recorded in ImageJ and subsequently analysed in Microsoft Excel. Spindle morphology was also analysed using Image J, all images were 3D rendered the maximum length and width of the spindle determined. Analysis of bivalent biorientation was done by semi-automated registering of kinetochore positions. ImageJ macros then determined the position and normal angle of the spindle equator and calculated the inter-kinetochore distance, alignment and angle of each bivalent at each timepoint.

### Statistical analysis

Chi-squared and analysis of variance (ANOVA) tests were performed using Prism software (GraphPad Software, Inc.). ANOVA was used with Tukey's *post hoc* test.

## Additional information

**How to cite this article:** Collins, J. K. *et al.* DNA damage induces a meiotic arrest in mouse oocytes mediated by the spindle assembly checkpoint. *Nat. Commun*. 6:8553 doi: 10.1038/ncomms9553 (2015).

## Supplementary Material

Supplementary Information Supplementary Figures 1-3

## References

[b1] Adriaens I., Smitz J. & Jacquet P. The current knowledge on radiosensitivity of ovarian follicle development stages. Hum. Reprod. Update 15, 359–377 (2009). 19151106 10.1093/humupd/dmn063

[b2] Meirow D., Epstein M., Lewis H., Nugent D. & Gosden R. G. Administration of cyclophosphamide at different stages of follicular maturation in mice: effects on reproductive performance and fetal malformations. Hum. Reprod. 16, 632–637 (2001). 11278209 10.1093/humrep/16.4.632

[b3] Kirk M. & Lyon M. F. Induction of congenital anomalies in offspring of female mice exposed to varying doses of X-rays. Mutat. Res. 106, 73–83 (1982). 7162529 10.1016/0027-5107(82)90191-9

[b4] Arnon J., Meirow D., Lewis-Roness H. & Ornoy A. Genetic and teratogenic effects of cancer treatments on gametes and embryos. Hum. Reprod. Update 7, 394–403 (2001). 11476352 10.1093/humupd/7.4.394

[b5] Suh E. K. *et al.* p63 protects the female germ line during meiotic arrest. Nature 444, 624–628 (2006). 17122775 10.1038/nature05337

[b6] Livera G. *et al.* p63 null mutation protects mouse oocytes from radio-induced apoptosis. Reproduction 135, 3–12 (2008). 18159078 10.1530/REP-07-0054

[b7] Marangos P. & Carroll J. Oocytes progress beyond prophase in the presence of DNA damage. Curr. Biol. 22, 989–994 (2012). 22578416 10.1016/j.cub.2012.03.063

[b8] Li X. M. *et al.* DNA topoisomerase II is dispensable for oocyte meiotic resumption but is essential for meiotic chromosome condensation and separation in mice. Biol. Reprod. 89, 118 (2013). 24048577 10.1095/biolreprod.113.110692

[b9] Damelin M. & Bestor T. H. The decatenation checkpoint. Br. J. Cancer 96, 201–205 (2007). 17211475 10.1038/sj.bjc.6603537 PMC2360007

[b10] Shaltiel I. A., Krenning L., Bruinsma W. & Medema R. H. The same, only different—DNA damage checkpoints and their reversal throughout the cell cycle. J. Cell Sci. 128, 607–620 (2015). 25609713 10.1242/jcs.163766

[b11] Lane S. I., Yun Y. & Jones K. T. Timing of anaphase-promoting complex activation in mouse oocytes is predicted by microtubule-kinetochore attachment but not by bivalent alignment or tension. Development 139, 1947–1955 (2012). 22513370 10.1242/dev.077040

[b12] Kolano A., Brunet S., Silk A. D., Cleveland D. W. & Verlhac M. H. Error-prone mammalian female meiosis from silencing the spindle assembly checkpoint without normal interkinetochore tension. Proc. Natl Acad. Sci. USA 109, E1858–E1867 (2012). 22552228 10.1073/pnas.1204686109 PMC3390881

[b13] Gui L. & Homer H. Spindle assembly checkpoint signalling is uncoupled from chromosomal position in mouse oocytes. Development 139, 1941–1946 (2012). 22513372 10.1242/dev.078352 PMC3347686

[b14] Sebestova J., Danylevska A., Novakova L., Kubelka M. & Anger M. Lack of response to unaligned chromosomes in mammalian female gametes. Cell Cycle 11, 3011–3018 (2012). 22871737 10.4161/cc.21398 PMC3442912

[b15] Nagaoka S. I., Hodges C. A., Albertini D. F. & Hunt P. A. Oocyte-specific differences in cell-cycle control create an innate susceptibility to meiotic errors. Curr. Biol. 21, 651–657 (2011). 21497085 10.1016/j.cub.2011.03.003 PMC3225230

[b16] McGuinness B. E. *et al.* Regulation of APC/C activity in oocytes by a Bub1-dependent spindle assembly checkpoint. Curr. Biol. 19, 369–380 (2009). 19249208 10.1016/j.cub.2009.01.064

[b17] Touati S. A. *et al.* Mouse oocytes depend on BubR1 for proper chromosome segregation but not for prophase I arrest. Nat. Commun. 6, 6946 (2015). 25897860 10.1038/ncomms7946 PMC4439927

[b18] Musacchio A. Spindle assembly checkpoint: the third decade. Philos. Trans. R. Soc. Lond. B Biol. Sci. 366, 3595–3604 (2011). 22084386 10.1098/rstb.2011.0072 PMC3203455

[b19] Foley E. A. & Kapoor T. M. Microtubule attachment and spindle assembly checkpoint signalling at the kinetochore. Nat. Rev. Mol. Cell Biol. 14, 25–37 (2013). 23258294 10.1038/nrm3494 PMC3762224

[b20] Lara-Gonzalez P., Westhorpe F. G. & Taylor S. S. The spindle assembly checkpoint. Curr. Biol. 22, R966–R980 (2012). 23174302 10.1016/j.cub.2012.10.006

[b21] Yuen W. S., Merriman J. A., O'Bryan M. K. & Jones K. T. DNA double strand breaks but not interstrand crosslinks prevent progress through meiosis in fully grown mouse oocytes. PLoS ONE 7, e43875 (2012). 22928046 10.1371/journal.pone.0043875 PMC3425511

[b22] Ma J. Y. *et al.* The effects of DNA double-strand breaks on mouse oocyte meiotic maturation. Cell Cycle 12, 1233–1241 (2013). 23518501 10.4161/cc.24311 PMC3674088

[b23] Dotiwala F., Harrison J. C., Jain S., Sugawara N. & Haber J. E. Mad2 prolongs DNA damage checkpoint arrest caused by a double-strand break via a centromere-dependent mechanism. Curr. Biol. 20, 328–332 (2010). 20096585 10.1016/j.cub.2009.12.033 PMC2811853

[b24] Eliezer Y., Argaman L., Kornowski M., Roniger M. & Goldberg M. Interplay between the DNA damage proteins MDC1 and ATM in the regulation of the spindle assembly checkpoint. J. Biol. Chem. 289, 8182–8193 (2014). 24509855 10.1074/jbc.M113.532739 PMC3961647

[b25] Kim E. M. & Burke D. J. DNA damage activates the SAC in an ATM/ATR-dependent manner, independently of the kinetochore. PLoS Genet. 4, e1000015 (2008). 18454191 10.1371/journal.pgen.1000015 PMC2265443

[b26] Nalepa G. *et al.* Fanconi anemia signaling network regulates the spindle assembly checkpoint. J. Clin. Invest. 123, 3839–3847 (2013). 23934222 10.1172/JCI67364 PMC3754252

[b27] Zachos G. *et al.* Chk1 is required for spindle checkpoint function. Dev. Cell 12, 247–260 (2007). 17276342 10.1016/j.devcel.2007.01.003 PMC7115955

[b28] Yang C. *et al.* The kinetochore protein Bub1 participates in the DNA damage response. DNA Repair (Amst.) 11, 185–191 (2012). 22071147 10.1016/j.dnarep.2011.10.018 PMC3758123

[b29] Terasawa M., Shinohara A. & Shinohara M. Canonical non-homologous end joining in mitosis induces genome instability and is suppressed by M-phase-specific phosphorylation of XRCC4. PLoS Genet. 10, e1004563 (2014). 25166505 10.1371/journal.pgen.1004563 PMC4148217

[b30] Giunta S., Belotserkovskaya R. & Jackson S. P. DNA damage signaling in response to double-strand breaks during mitosis. J. Cell Biol. 190, 197–207 (2010). 20660628 10.1083/jcb.200911156 PMC2930281

[b31] Cesare A. J. Mitosis, double strand break repair, and telomeres: A view from the end: How telomeres and the DNA damage response cooperate during mitosis to maintain genome stability. Bioessays 36, 1054–1061 (2014). 25171524 10.1002/bies.201400104 PMC4270212

[b32] Bakhoum S. F., Kabeche L., Murnane J. P., Zaki B. I. & Compton D. A. DNA-damage response during mitosis induces whole-chromosome missegregation. Cancer Discov. 4, 1281–1289 (2014). 25107667 10.1158/2159-8290.CD-14-0403 PMC4221427

[b33] Orthwein A. *et al.* Mitosis inhibits DNA double-strand break repair to guard against telomere fusions. Science 344, 189–193 (2014). 24652939 10.1126/science.1248024

[b34] Scarpato R. *et al.* Kinetics of nuclear phosphorylation (gamma-H2AX) in human lymphocytes treated in vitro with UVB, bleomycin and mitomycin C. Mutagenesis 28, 465–473 (2013). 23696313 10.1093/mutage/get024

[b35] de Feraudy S., Revet I., Bezrookove V., Feeney L. & Cleaver J. E. A minority of foci or pan-nuclear apoptotic staining of gammaH2AX in the S phase after UV damage contain DNA double-strand breaks. Proc. Natl Acad. Sci. USA 107, 6870–6875 (2010). 20351298 10.1073/pnas.1002175107 PMC2872460

[b36] Lobrich M. *et al.* gammaH2AX foci analysis for monitoring DNA double-strand break repair: strengths, limitations and optimization. Cell Cycle 9, 662–669 (2010). 20139725 10.4161/cc.9.4.10764

[b37] Holt J. E., Lane S. I. & Jones K. T. The control of meiotic maturation in mammalian oocytes. Curr. Top. Dev. Biol. 102, 207–226 (2013). 23287034 10.1016/B978-0-12-416024-8.00007-6

[b38] Reis A., Chang H. Y., Levasseur M. & Jones K. T. APCcdh1 activity in mouse oocytes prevents entry into the first meiotic division. Nat. Cell Biol. 8, 539–540 (2006). 16715549 10.1038/ncb1406 PMC2435241

[b39] Wiersma A. *et al.* Phosphodiesterase 3 inhibitors suppress oocyte maturation and consequent pregnancy without affecting ovulation and cyclicity in rodents. J. Clin. Invest. 102, 532–537 (1998). 9691090 10.1172/JCI2566 PMC508914

[b40] Jones K. T. & Lane S. I. Molecular causes of aneuploidy in mammalian eggs. Development 140, 3719–3730 (2013). 23981655 10.1242/dev.090589

[b41] Deng M., Suraneni P., Schultz R. M. & Li R. The Ran GTPase mediates chromatin signaling to control cortical polarity during polar body extrusion in mouse oocytes. Dev. Cell 12, 301–308 (2007). 17276346 10.1016/j.devcel.2006.11.008

[b42] Kouznetsova A., Lister L., Nordenskjold M., Herbert M. & Hoog C. Bi-orientation of achiasmatic chromosomes in meiosis I oocytes contributes to aneuploidy in mice. Nat. Genet. 39, 966–968 (2007). 17618286 10.1038/ng2065

[b43] Yun Y., Lane S. I. & Jones K. T. Premature dyad separation in meiosis II is the major segregation error with maternal age in mouse oocytes. Development 141, 199–208 (2014). 24346700 10.1242/dev.100206 PMC3913075

[b44] Lane S. I. & Jones K. T. Non-canonical function of spindle assembly checkpoint proteins after APC activation reduces aneuploidy in mouse oocytes. Nat. Commun. 5, 3444 (2014). 24637522 10.1038/ncomms4444

[b45] Kitajima T. S., Ohsugi M. & Ellenberg J. Complete kinetochore tracking reveals error-prone homologous chromosome biorientation in mammalian oocytes. Cell 146, 568–581 (2011). 21854982 10.1016/j.cell.2011.07.031

[b46] Hached K. *et al.* Mps1 at kinetochores is essential for female mouse meiosis I. Development 138, 2261–2271 (2011). 21558374 10.1242/dev.061317

[b47] Tsurumi C., Hoffmann S., Geley S., Graeser R. & Polanski Z. The spindle assembly checkpoint is not essential for CSF arrest of mouse oocytes. J. Cell Biol. 167, 1037–1050 (2004). 15611331 10.1083/jcb.200405165 PMC2172623

[b48] Homer H. A. *et al.* Mad2 prevents aneuploidy and premature proteolysis of cyclin B and securin during meiosis I in mouse oocytes. Genes Dev. 19, 202–207 (2005). 15655110 10.1101/gad.328105 PMC545877

[b49] Howlett S. K. & Bolton V. N. Sequence and regulation of morphological and molecular events during the first cell cycle of mouse embryogenesis. J. Embryol. Exp. Morphol. 87, 175–206 (1985). 4031752

[b50] Lord T. & Aitken R. J. Oxidative stress and ageing of the post-ovulatory oocyte. Reproduction 146, R217–R227 (2013). 23950493 10.1530/REP-13-0111

[b51] Meraldi P., Draviam V. M. & Sorger P. K. Timing and Checkpoints in the Regulation of Mitotic Progression. Dev. Cell 7, 45–60 (2004). 15239953 10.1016/j.devcel.2004.06.006

[b52] Rodriguez-Bravo V. *et al.* Nuclear pores protect genome integrity by assembling a premitotic and Mad1-dependent anaphase inhibitor. Cell 156, 1017–1031 (2014). 24581499 10.1016/j.cell.2014.01.010 PMC3947552

[b53] Polanski Z. Spindle assembly checkpoint regulation of chromosome segregation in mammalian oocytes. Reprod. Fertil. Dev. 25, 472–483 (2013). 22951024 10.1071/RD12145

[b54] Jones K. T. & Lane S. I. Chromosomal, metabolic, environmental, and hormonal origins of aneuploidy in mammalian oocytes. Exp. Cell Res. 318, 1394–1399 (2012). 22394508 10.1016/j.yexcr.2012.02.012

[b55] Tachibana-Konwalski K. *et al.* Spindle assembly checkpoint of oocytes depends on a kinetochore structure determined by cohesin in meiosis I. Curr. Biol. 23, 2534–2539 (2013). 24291092 10.1016/j.cub.2013.10.052 PMC3898714

[b56] Skoufias D. A., Lacroix F. B., Andreassen P. R., Wilson L. & Margolis R. L. Inhibition of DNA decatenation, but not DNA damage, arrests cells at metaphase. Mol. Cell 15, 977–990 (2004). 15383286 10.1016/j.molcel.2004.08.018

[b57] Tomasini R. *et al.* TAp73 knockout shows genomic instability with infertility and tumor suppressor functions. Genes Dev. 22, 2677–2691 (2008). 18805989 10.1101/gad.1695308 PMC2559903

[b58] Wilhelm M. T. *et al.* Isoform-specific p73 knockout mice reveal a novel role for delta Np73 in the DNA damage response pathway. Genes Dev. 24, 549–560 (2010). 20194434 10.1101/gad.1873910 PMC2841333

[b59] Tomasini R. *et al.* TAp73 regulates the spindle assembly checkpoint by modulating BubR1 activity. Proc. Natl Acad. Sci. USA 106, 797–802 (2009). 19139399 10.1073/pnas.0812096106 PMC2621249

