# Supplementary material for: DNA damage induces a meiotic arrest in mouse oocytes mediated by the spindle assembly checkpoint
Source: Nat Commun. 2015 Nov 2;6:8553. doi: 10.1038/ncomms9553 (PMC4659839; doi:10.1038/ncomms9553)
Supplement: Supplementary Information — Supplementary Figures 1-3 [file ncomms9553-s1.pdf]

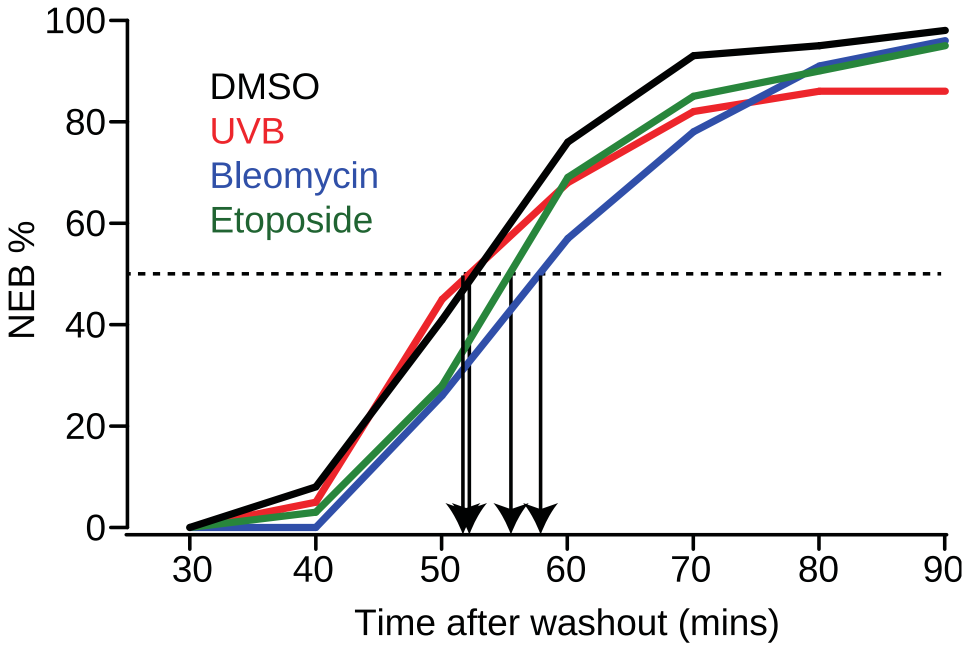


**Supplementary Figure 1. Lack of effect of DNA damage on the timing of nuclear envelope breakdown.**

The timing of NEB in a population of GV oocytes treated either with 0.1% DMSO (n = 59); etoposide (25 μg $ml^{-1}$; n = 39); bleomycin (1 μM; n = 23); or UVB (15 s; n = 22). The vertical arrows indicate the times at which 50% of oocytes have undergone NEB (NEB-50%). There was at most only a 10 min difference in the timing of NEB-50% between oocytes from the difference groups.

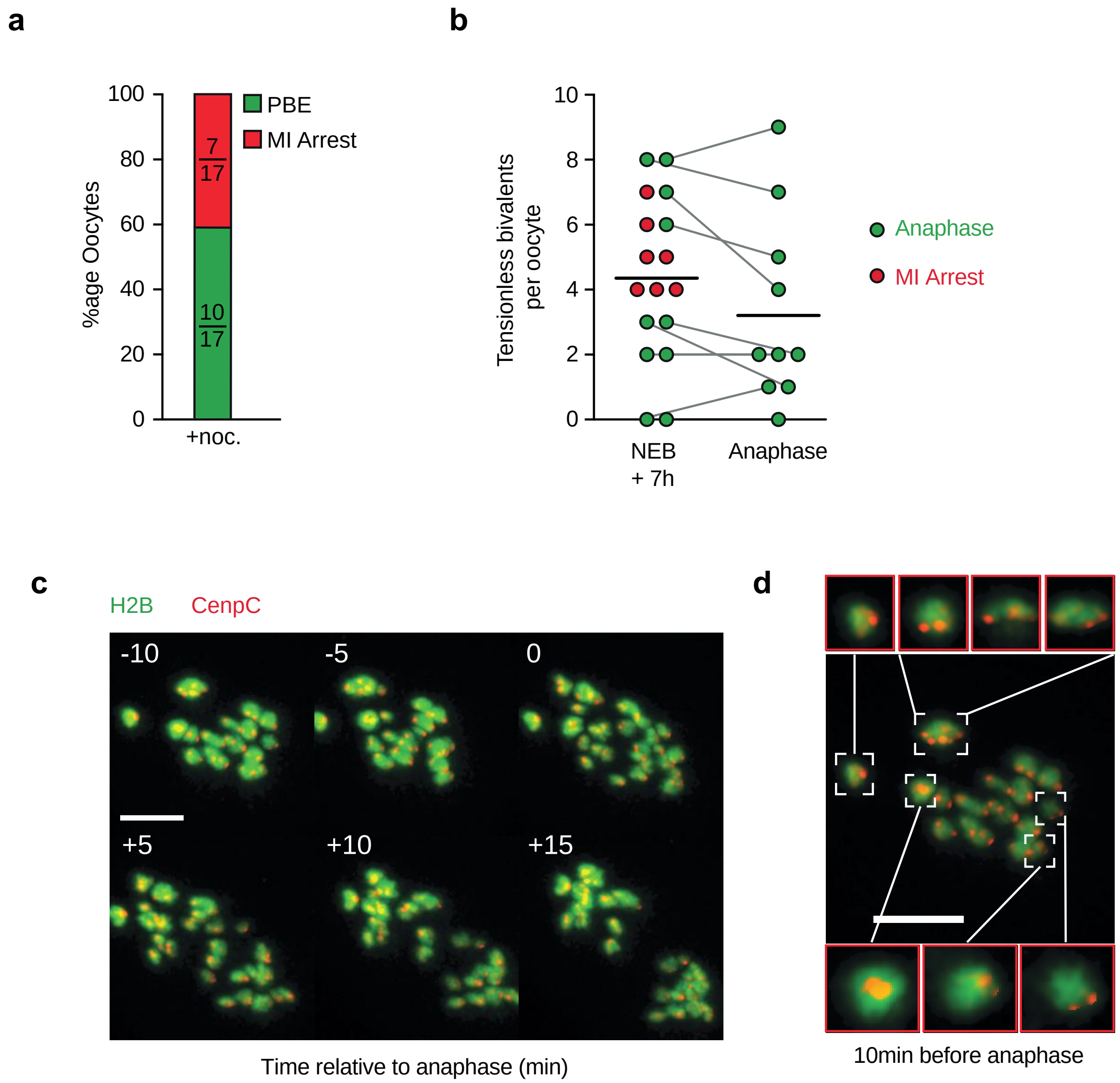


**Supplementary Figure 2. A lack of tension across multiple bivalents fails to arrest oocytes in MI.**

(**a**) Nocodazole (25 nM) addition to culture media throughout maturation permitted 59% PBE. (**b**) Number of bivalents without apparent tension across their sister kinetochore pairs at 7 h after NEB, or in the frame prior to anaphase, in oocytes matured in the presence of 25 nM nocodazole, expressing CenpC-GFP and H2B-mCherry. Black horizontal line indicates mean, gray lines connect data points from the same oocyte. (**c,d**) Bivalents imaged during anaphase from a

representative oocyte (c), and an enlarged image (d) 10 min before anaphase. These images show an oocyte about to undergo anaphase with 7 bivalents that are not properly biorientated, and appear to have little tension across their sister kinetochore pairs (insets). Scale bar represents 10 μm.

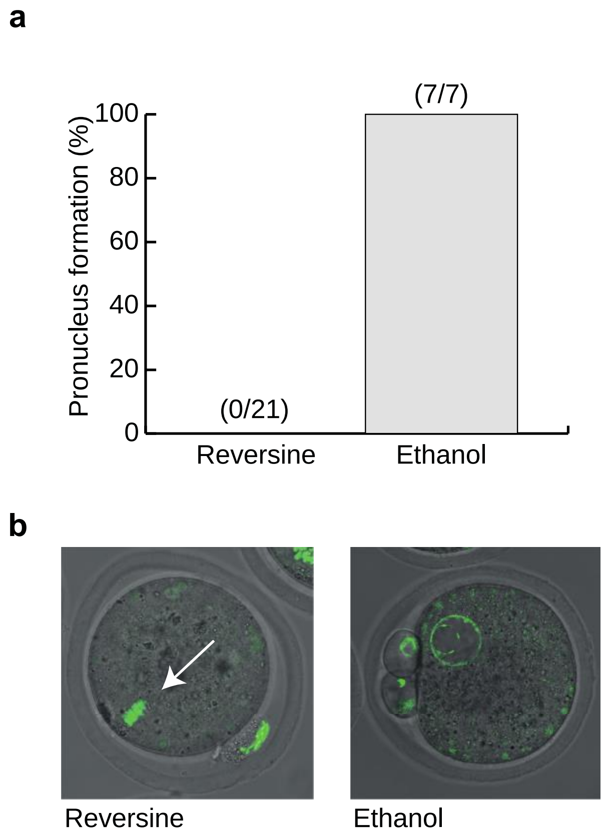


**Supplementary Figure 3. Reversine does not have any effect on the metaphase arrest of eggs.**

(**a**) Percentage of eggs forming either a second polar body or pronuclei at 6 h following treatment of metaphase II eggs with the parthenogenetic agent ethanol (7% ethanol for 7 min at room temperature) or reversine (100 nM). (**b**) Representative images of eggs treated with reversine or ethanol. Eggs are still arrested at metaphase following reversine (arrow marks metaphase plate), but not with ethanol, where activated eggs possess both a second polar body and a pronucleus.
